# Impact of aging on TREM-1 responses in the periodontium: a cross-sectional study in an elderly population

**DOI:** 10.1186/s12879-016-1778-6

**Published:** 2016-08-19

**Authors:** Veli Özgen Öztürk, Georgios N. Belibasakis, Gülnur Emingil, Nagihan Bostanci

**Affiliations:** 1 Department of Periodontology, School of Dentistry, Adnan Menderes University, Aydın, Turkey; 2 Department of Dental Medicine, Karolinska Institute, Stockholm, Sweden; 3 Department of Periodontology, School of Dentistry, Ege University, Izmir, Turkey

**Keywords:** sTREM-1, Aging, Gingival crevicular fluid, Subgingival plaque, Elderly

## Abstract

**Background:**

Aging is associated with altered immune response, which increases susceptibility to infections. sTREM-1 is involved in the amplification of the inflammatory response to bacterial infection. The present cross-sectional study aims to investigate local sTREM-1 levels in gingival crevicular fluid (GCF) as well as key periodontal pathogen levels in the subgingival plaque in an elderly cohort with periodontal health, gingivitis, and chronic periodontitis (CP).

**Methods:**

Subjects were 51 systemically healthy, elderly individuals (mean age, 68 ± 4.5 years) who had undergone full-mouth periodontal examinations. Subgingival plaque and GCF samples were collected from the healthy sites of participants without periodontal disease (*n* = 17), the sites with gingival inflammation from patients with gingivitis (*n* = 19), and the periodontitis sites of patients with CP (*n* = 15). GCF volumes were measured by an electronic impedance device, and total protein levels were assessed by a flouremetric assay. sTREM-1 levels in GCF were measured by enzyme-linked immunosorbent assay. The subgingival plaque total bacteria, *Porphyromonas gingivalis*, *Tannerella forsythia*, *Treponema denticola*, *Fusobacterium nucleatum*, *and Prevotella intermedia* levels were determined by quantitative real-time polymerase chain reaction. Statistical analysis was performed using nonparametric methods.

**Results:**

GCF volume, total protein concentrations, and sTREM-1 levels in GCF were similar among the groups (*p* > 0.05). Significantly higher *T. forsythia* levels were observed in subgingival plaque samples harvested from patients with gingivitis and CP, than in those from healthy participants (*p* < 0.05). However, the subgingival levels of the other four periodontal pathogens and total bacteria were not statistically different among the groups (*p* > 0.05).

**Conclusions:**

Our findings suggest that there are no differences in GCF volume, total protein, and sTREM-1 levels between healthy and periodontally diseased elderly adults. We found only limited differences in the studied subgingival microbial profile. This finding indicates an already deregulated, local inflammatory response in this elderly cohort, on which bacterial biofilm challenge may have a limited further impact.

## Background

Periodontal diseases in their severe form affect approximately 10 % of the global population, and their direct treatment poses a significant annual burden on national health systems. They are characterized by localized inflammatory destruction of periodontal tissues, occur as a result of subgingival biofilm formation [1]. The host immune response against the offending microorganisms is a key factor to subsequent periodontal tissue destruction [2, 3]. Considerable individual variation exists in microbial challenge and host responses to periodontal bacteria [4–6], such as *Porphyromonas gingivalis*, *Tannerella forsythia*, and *Treponema denticola* [7, 8]*.* Host-related factors, such as aging, can also impair the periodontal host response [9].

The World Health Organisation defines an elderly person as one who is 65 years and older [10]. Many physical changes, such as reduction in bone mass, compromised immune system, and cancer, occur with increasing age [11]. Periodontal disease is one such change, as it is a common chronic inflammatory disease among the elderly [11]. The prevalence of mild to moderate periodontitis among the elderly is high [11, 12]. Inflammatory mediator levels generally exhibit age-related changes, even in the absence of acute infection or other physiologic stress [13]. A significant relationship exists between aging, inflammation, response to infection, and progression of chronic, inflammatory diseases [14]. Although periodontal disease is a common chronic, inflammatory disease that occurs during an individual’s lifetime, its relationship with aging has not been fully elucidated.

TREM-1, a receptor belonging to the immunoglobulin superfamily, is produced and released during the course of local and systemic infections, such as septic shock and pneumonia [15–18]. To date, the role of TREM-1 in innate immunity is postulated to be the regulation of the inflammatory response magnitude to bacterial challenge, which leads to increased proinflammatory cytokine production [19, 20]. During infection, TREM-1 is released from the cell surface as its soluble sTREM-1 form. Because of this property, sTREM-1 has been proposed as a biomarker of systemic inflammation in systemic sepsis, arthritis, pulmonary infection, pancreatitis, and inflammatory bowel disease [17, 18, 21–23].

A number of recent in vitro studies have shown that *P. gingivalis* can stimulate TREM-1 production in monocytes and neutrophils, promoting a shift from its cell-membrane, bound form to sTREM-1, which is also accompanied by propagation of proinflammatory cytokine production [24–26]. We have shown in other studies that local and systemic sTREM-1 levels are elevated in patients with periodontitis compared to healthy individuals [27, 28].

Nevertheless, the role of aging in periodontal inflammation or the role of TREM-1 in elderly participants has not been clearly established. It is worth noting that TREM-1-induced polymorphonuclear neutrophil functions, following TREM-1 engagement, exhibit an impaired response when obtained from elderly donors [28]. In line with this report, another study showed that TREM-1 expression was elevated in macrophages obtained from aged mice [29].

Hence, the hypothesis of the present study is that the association between GCF sTREM-1 levels and periodontal status, or subgingival microbiological parameters, is impaired due to chronic exposure in an elderly population. The null hypothesis is that age does not affect these parameters in the gingival crevice or periodontal pocket. Therefore, the aim of the present study was to assess local sTREM-1 levels in GCF and key periodontal pathogen levels in the subgingival plaque of an elderly cohort with periodontal health, gingivitis and CP.

## Methods

### Study population and clinical examination

This study includes 51 elderly individuals who were recruited from the Department of Periodontology, School of Dentistry, Ege University, Izmir, Turkey, between April and September 2013. The study was approved by the Ethics Committee of the Medical Faculty of Ege University (ethics approval number: 11–12.1/11) and was conducted according to the guidelines of the Declaration of Helsinki. Written, informed consent was obtained from each of the 51 participants. Inclusion criteria included being: age 65 or older, systemically healthy, and non-smoker. Exclusion criteria included: cardiovascular or respiratory diseases, diabetes mellitus, HIV infection, immunosuppressive chemotherapy, and current acute infection. None of the participants had received antibiotics or other medications or periodontal treatment within the past four-months.

All participants were evaluated clinically and radiographically by a single, calibrated examiner experienced in evaluating participants in clinical trials (VÖÖ). Full-mouth clinical periodontal parameters were recorded, including measurement of probing-pocket depth and clinical attachment level at six sites around each tooth with a manual probe [Williams probe]. Measurements in third molars were excluded. Also recorded were PI [30] and dichotomous measurement of BOP. Patient selection was conducted according to the criteria proposed by the 1999 International World Workshop for a Classification of Periodontal Disease and Conditions [1]. Individuals of the control group had >15 teeth, no history of periodontitis, no sites with >3 mm PPD, and >1 mm CAL as well as no radiographic evidence of alveolar bone loss. Patients of the gingivitis group exhibited no sites with alveolar bone loss and had varying degrees of gingival inflammation, but had no CAL >2 mm and full-mouth BOP >50 %. Members of the periodontitis group had moderate to severe alveolar bone loss and at least four nonadjacent teeth with sites of CAL of ≥6 mm, a PPD ≥5 mm, and a full-mouth BOP >50 %.

### Collection of GCF samples

After being selected for the study, participants were recalled for GCF sampling with periopaper collection strips (OraFlow Inc., Smithtown, NY, USA). The GCF samples were collected from the two mesiobuccal aspects of a single-rooted tooth with ≥5 mm probing depth with BOP in the periodontitis group (*n* = 15, with mean PPD: 5.46 ± 0.51, CAL: 6.40 ± 0.98, PI: 2.52 ± 0.20), while 2 GCF samples were collected from two mesiobuccal aspects of a single-rooted tooth with probing depth up to 3 mm with BOP in the gingivitis group (*n* = 19, with mean PPD: 2.68 ± 0.47, CAL: 2.68 ± 0.56, PI: 2.57 ± 0.50). In the healthy group two GCF samples were collected from the mesiobuccal aspect of single-rooted teeth exhibiting probing-pocket depth up to 3 mm without BOP (*n* = 17, with mean PPD: 2.00 ± 0.61, CAL: 2.00 ± 0.61, PI: 0.17 ± 0.39). The volume of GCF absorbed in each strip (OraFlow Inc. Smithtown, NY, USA) was determined by electronic impedance (Periotron 8000; Proflow, Inc., Amityville, NY, USA). Immediately afterwards, the collected paper strips were directly placed into microcentrifuge tubes and stored at 80 °C until needed for laboratory analysis.

### Determination of total protein concentrations in GCF samples

On the day of analysis, the GCF samples were eluted in 420 μL of phosphate-buffered saline (pH 7.2), containing EDTA-free Protease Inhibitor Cocktail (Roche Applied Science, Basel, Switzerland) by centrifugation at 5000 × g for 10 min at 4 °C. The total protein content of GCF was quantified with the Qubit Protein Assay Kit (Thermo Fisher Scientific, Zug, Switzerland) according to manufacturer’s instructions. Briefly, 3 assay tubes were prepared for standards and one assay tube per sample. Qubit working solution was prepared by diluting the Qubit reagent in buffer. Then 10 μL sample or standard were added to assay tubes, which were incubated for 15 min at room temperature. Samples were incubated for 15 min. Then, the tubes were read in the Qubit® 2.0 Flourometer (Thermo Fisher Scientific, Zug, Switzerland).

### Determination of sTREM-1 in GCF samples

sTREM-1 levels in GCF were measured using an ELISA kit (DuoSet, R&D Systems, Abingdon, UK) according to manufacturer‘s instructions with some modifications. Briefly, 100 μL of standards or test samples were added to microplates in duplicate, incubated for 2 h at room temperature, followed by removal of the liquid from each well. A biotin-conjugated antibody specific for sTREM-1 and avidin conjugated to horseradish peroxidase was added to each well, incubated for 2 h at room temperature, and the unbound antibody was removed by washing in PBS twice for 2 min on a stirring plate. Then, tetramethylbenzidine was added as a substrate solution to each well and incubated for 20 min at room temperature, after which the reaction was stopped by the addition of 1 M sulfuric acid solution. The absorbance was measured at 450 nm using a microplate reader (Epoch, BioTek, Luzern, Switzerland), with a wavelength correction set at 570 nm to subtract background. The concentration of sTREM-1 in the samples was then determined by comparing the absorbance of the samples with the standard curve generated by the measurement of the concentrations of recombinant human sTREM-1. The results were presented in two ways: (i) dividing total amount of sTREM-1 recovered in 420 μL PBS by the volume of the sample as measured by Periotron, and expressed as pg/μL) and (ii) calibrating against total protein amount, as measured by a fluorometric assay and expressed as pg/mg protein.

### Collection and processing of subgingival plaque samples

Approximately 15 min after GCF collection, subgingival plaque samples were collected from the four mesiobuccal surfaces of the preselected, single-rooted teeth using two standardized ♯ 30 sterile paper points. One point was inserted at a 45° angle and the other parallel to the long axis of the tooth and left place for 10 s. The paper points were then frozen at −70 ° C until further use. Subgingival plaque samples were collected from the mesiobuccal surface aspects of a single-rooted tooth with ≥5 mm probing depth with BOP in the CP group (*n* = 15, with mean PPD: 5.65 ± 0.40, CAL: 6.07 ± 0.32, PI: 2.40 ± 0.25), while the other samples were collected the from 4 mesiobuccal surfaces of a single-rooted tooth with probing depth up to 3 mm with BOP in the gingivitis group (*n* = 19, with mean PPD: 2.52 ± 0.56, CAL: 2.52 ± 0.56, PI: 2.45 ± 0.50). In the healthy group, the samples were collected from the mesiobuccal aspect of single-rooted teeth exhibiting probing-pocket depth up to 3 mm without BOP (*n* = 17, with mean PPD: 2.10 ± 0.42, CAL: 2.10 ± 0.42, PI: 0.16 ± 0.22). For DNA extraction, the collected subgingival plaque samples were processed using a commercially available kit (GenElute Bacterial Genomic DNA Kit, Sigma-Aldrich, Buchs, Switzerland), and the concentration of resulting DNA was measured spectrophotometrically (NanoDrop 1000, Thermo Fisher Scientific, Wohlen, Switzerland).

### Bacterial quantification by qPCR

Selected putative, periodontal pathogens and total bacterial load in the subgingival plaque were detected as described earlier [31]. Briefly, DNA amplification and detection were performed in a qPCR system (StepOnePlus, Applied Biosystems Life Technologies, Basel, Switzerland), using master mix SYBR Green Master Mix (Applied Biosystems Life Technologies, Basel, Switzerland) for the amplification reaction. Each reaction was performed in a total of 20 μL, including the species-specific oligonucleotide primers and 10 ng DNA extracted from subgingival plaque samples, according to manufacturer’s guidelines as described earlier [27]. Bacterial DNA of known amounts (1.0–0.0001 ng) was extracted from pure cultures of the taxa under investigation for the purpose of generating standard curves to estimate number of copies of each species in the subgingival plaque. Briefly, *P. gingivalis* (ATCC 33277 T; OMZ 925) was grown anaerobically on Columbia Blood Agar (CBA) agar plates for 3–4 days at 37 °C, followed by anaerobic subculturing for 2–3 days at 37 °C in broth. *T. forsythia* (OMZ 1047), *T. denticola* (ATCC 35405; OMZ 661), *P. intermedia* (OMZ 278), *F. nucleatum* (OMZ 598), were maintained in liquid cultures as described earlier [32]. DNA was extracted from these cultures and measured as indicated above. The lowest detection limits for the qPCR assays were defined by the cycle, threshold signal corresponding to the lowest DNA amount used for generating the standard curve (0.0001 ng). The theoretical bacterial numbers in each sample were calculated on the basis of measured amount of DNA and the estimated genome weight as described earlier [31].

### Statistical analyses

Statistical analyses were conducted using a statistical software package (GraphPad Software, La Jolla California USA). The minimum sample size was calculated in healthy and diseased groups by accepting a power of 90 % and a *P*-value of 5 %. A power calculation suggested that the minimum sample size required was 13 participants for each group. Distribution of data was evaluated by the normality test. Comparisons among the non-normally-distributed variables, such as GCF sTREM-1 and subgingival bacterial levels, were performed by the Kruskal–Wallis one-way ANOVA and Dunn’s test. Clinical parameters and demographic data were normally distributed and assessed by ANOVA. *P* < 0.05 was considered statistically significant.

## Results

### Clinical findings

Demographic data and full-mouth clinical findings are provided in Table 1. Age and gender were similar among groups. Full-mouth mean PPD, CAL, PI, and BOP scores were significantly higher in the CP and gingivitis groups than in the healthy group (*P* < 0.0001) (Table 1). In contrast, the number of existing teeth was similar among groups.

**Table 1:** Full-mouth clinical findings of the study groups (*n* = 51)

Clinical Parameters	Healthy Group (*n* = 17)	Gingivitis Group (*n* = 19)	Chronic Periodontitis Group (*n* = 15)
Gender	7 F-10 M	8 F-11 M	9 F-6 M
Age	68.53 ± 4.57	68.47 ± 5.52	68.40 ± 3.13
Probing-pocket depth (mm)	1.97 ± 0.31	2.80 ± 0.18*	4.66 ± 0.22**
Clinical attachment level (mm)	1.98 ± 0.30	2.81 ± 0.18*	5.23 ± 0.31**
Plaque index	1.12 ± 0.32	2.29 ± 0.23*	2.52 ± 0.20*
Bleeding on probing (%)	1.29 ± 3.67	51.42 ± 13.06*	70.27 ± 11.20*
Number of teeth	24.17 ± 2.85	21.68 ± 1.91	21.93 ± 1.90
Number of single-rooted teeth	18.24 ± 2.33	17.53 ± 1.80	16.60 ± 1.76

The values represent the mean ± standard deviation (SD) of the studied clinical parameters

F: Female; M: Male

*Significant differences from the healthy group (*p* < 0.05)

**Significant differences from the healthy and gingivitis groups (*p* < 0.05)

### Microbiological findings in subgingival plaque

**Fig. 1:**
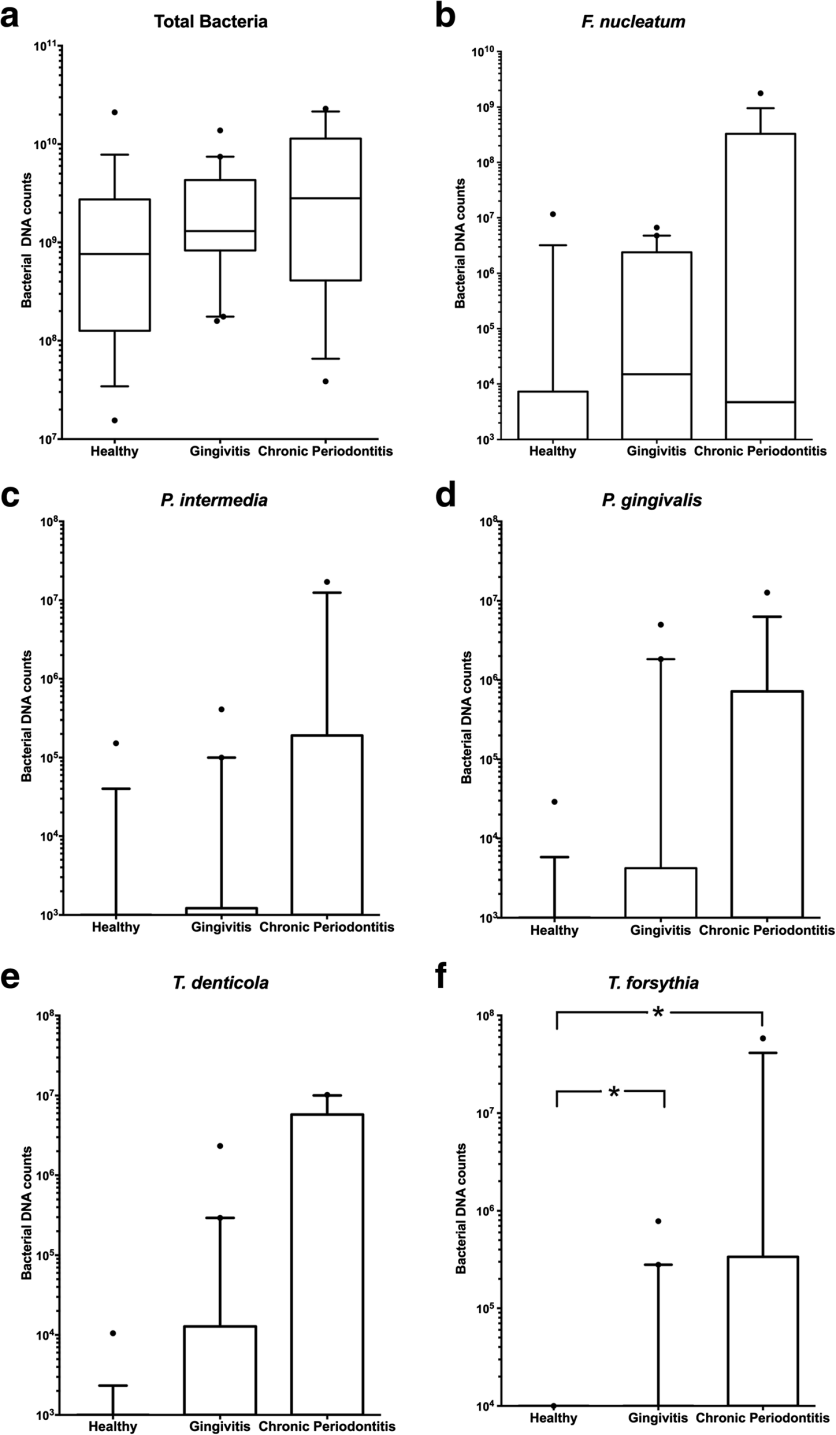
Oral microbiota levels in the subgingival plaque from elderly individuals with periodontal health (*n* = 17), gingivitis (*n* = 19), and chronic periodontitis (*n* = 15). The individual values represent bacterial counts per sample in the subgingival plaque (**a**) Total bacterial counts, (**b**) *F. nucleatum*, (**c**) *P. intermedia*, (**d**) *P. gingivalis*, (**e**) *T. denticola*, and (**f**) *T. forsythia*. The horizontal lines in the middle of the box represent the median and whiskers are drawn down to the 10^th^ through 90^th^ percentiles. The points below and above the whiskers are drawn as individual dots

Subgingival plaque samples were assessed by qPCR for five individual species and total bacterial counts. Frequencies of detection of species and bacterial counts are shown in Table 2. There were no differences in total bacterial levels among the three groups, with mean counts in the health group: 2.4 × 10^9^ ± 5.0 × 10^9^, in the gingivitis group: 2.8 × 10^9^ ± 3.3 × 10^9^, and in the CP group: 7.2 × 10^9^ ± 7.7 × 10^9^ (*P* > 0.05) (Fig. 1a). *P. gingivalis*, *T. denticola*, *F. nucleatum*, *and P. intermedia* in the subgingival plaque were also similar among the three groups (*P* >0.05) (Fig. 1b, c, d, and e). *T. forsythia* levels in the subgingival plaque were significantly higher in the CP group (by 6.5 × 10^5^-fold) and gingivitis group (by 6.4 × 10^3^-fold) than in the healthy group (*p* < 0.05) (Fig. 1f).

**Table 2:** Detection of periodontal pathogens in subgingival plaque of the elderly in healthy and diseased groups (*n* = 51)

Species	Healthy Group	Gingivitis Group	Chronic Periodontitis Group
*n*	17	19	15
*F. nucleatum* *n* (%)	8 (47)	12 (63)	8 (53)
*P. intermedia* *n* (%)	4 (23)	9 (47)	7 (46)
*P. gingivalis* *n* (%)	1 (5)	7 (36)	6 (40)
*T. denticola* *n* (%)	3 (17)	7 (36)	6 (40)
*T. forsythia* *n* (%)	1 (5)	7 (36)	7 (46)

### Analysis of GCF volume and total protein analysis

**Fig. 2:**
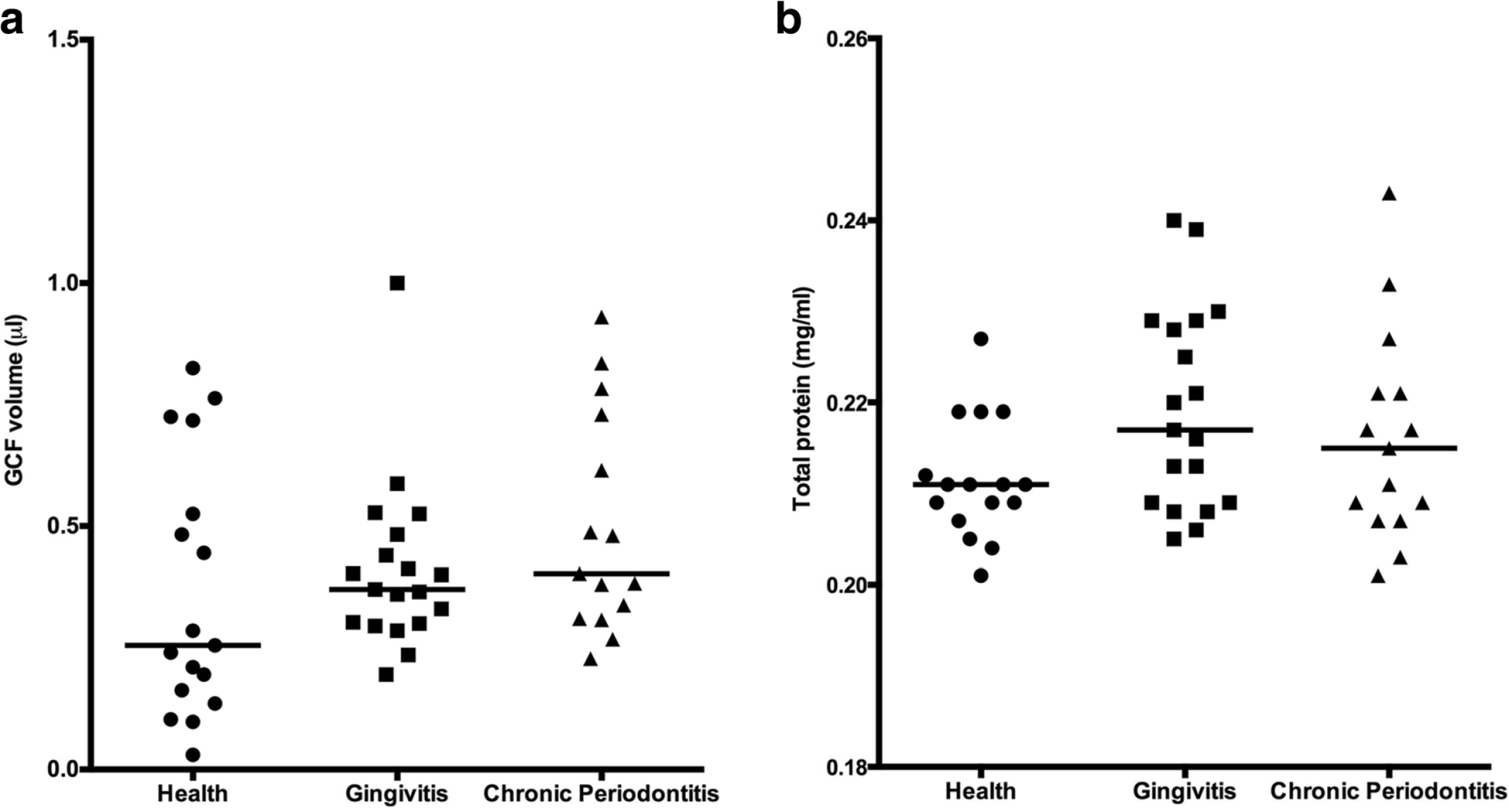
The inflammatory characteristics of the GCF samples from elderly patients with or without periodontal disease. GCF volume (**a**) was measured using a calibrated Periotron 8000, and then the readings were converted to an actual volume (μL) by reference to the standard curve. GCF total protein concentrations (**b**) were measured with a fluorometric assay. Horizontal lines show median values

The mean ± SEM value of GCF volume was 0.36 ± 0.26 μL in the healthy group, 0.41 ± 0.17 μL in the gingivitis group, and 0.49 ± 0.22 μL in the CP group (Fig. 2a). The volume of the collected GCF samples was not significantly different between the three groups (*P* > 0.05). In a similar manner, although the CP group had a 1.4-fold higher total protein content than the healthy group, that difference did not reach significance (*P* > 0.05) The mean ± SEM values were in the healthy group: 303.7 ± 105.30 mg/mL, gingivitis group: 370.26 ± 263.97 mg/mL, and CP group: 448 ± 93.18) (Fig. 2b).

### Analysis of sTREM-1 Levels in GCF

**Fig. 3:**
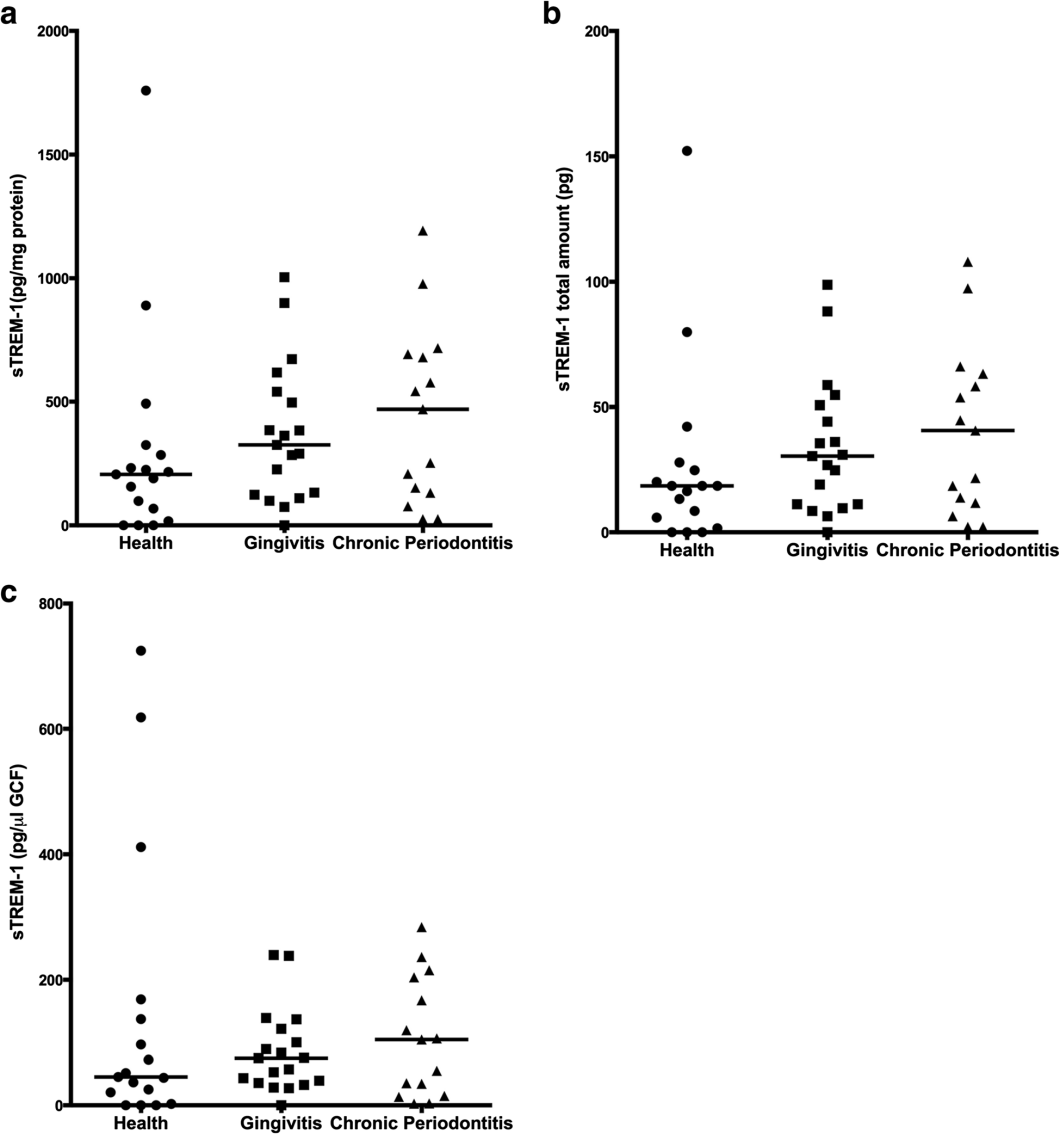
Distribution of sTREM-1 levels in GCF from healthy sites (*n* = 17), gingivitis-affected sites (*n* = 19), and periodontitis-affected sites of patients with CP (*n* = 15). sTREM-1 levels in the GCF samples were determined by ELISA. Individual values represent sTREM-1 as pg/mg of protein after normalization of the ELISA read-outs to total protein levels (**a**), sTREM-1 total amounts (pg) (**b**), or sTREM-1 concentrations (pg/μL) calibrated against corresponding GCF volume (**c**). Horizontal lines indicate the median values

Concentrations of sTREM-1 in GCF were expressed as total amounts (pg) (Fig. 3a) or pg/μL after normalizing total amounts to GCF volume (Fig. 3b) or as pg/mg of protein after normalization of the ELISA read-outs to total protein levels. sTREM-1 was not detected in three of 17 healthy participants and 1 of 19 gingivitis patients. sTREM-1 amounts in GCF (mean ± SEM) were 25.86 ± 9.27 pg in the healthy sites, 33.43 ± 6.39 pg in the gingivitis sites, and 40.56 ± 8.71 pg in the CP sites. However, none of the differences among the 3 groups were significant. Additionally, sTREM-1 concentrations either normalized by GCF volume (mean ± SEM, healthy group: 144 ± 54.00 pg/μL, gingivitis group: 85.05 ± 15.24 pg/μL, and CP group: 106.3 ± 24.40 pg/μL) or by total protein content did not significantly differ among the groups. The resulting sTREM-1 concentrations were calculated by dividing sTREM-1 amount values by volume of each GCF sample individually. However, none of the differences among any of the groups proved to be statistically significant.

## Discussion

Aging can alter immune cell functions that can greatly influence bacterial clearance and wound healing in any given site [33, 34], thus contributing to an increase in the occurrence of infections, including periodontitis, in the elderly population. Although prevalence of periodontal disease increases with age, the underlying factors for the increase remain unclear. The present cross-sectional study evaluated the levels of sTREM-1, an amplifier of inflammation [35], in GCF of elderly patients with gingivitis and CP and in individuals with clinically healthy periodontium. Recently, one study showed that patients with periodontitis have elevated sTREM-1 levels in the saliva and serum [36] or in the GCF, and that sTREM-1 levels increase with the severity of the disease [27, 37]. Additionally, sTREM-1 levels were positively associated with the presence of putative, periodontal pathogens present in the corresponding subgingival plaque samples [27]. To the best of our knowledge, the present study is the first to provide data on GCF of sTREM-1 in patients with CP and gingivitis and in systemically healthy elderly individuals. Interestingly, GCF sTREM-1 levels in elderly patients with CP and gingivitis were comparable with those in periodontally healthy individuals, as there were no differences among the groups. The lack of a statistically significant difference could potentially be explained by the aging process itself. In other terms, by a potential loss of the capacity of aged gingival tissue to respond to microbial challenge in a pathophysiologic fashion [38]. In support of this finding hinting for a reduced response of aged gingival tissues to microbial challenge, earlier studies showed that a number of monocyte/macrophage functions become compromised with age, including chemotaxis, phagocytosis, production of reactive oxygen species, and induction of certain cytokine responses [39, 40]. The effects of aging on periodontal tissues may be reflected as biomolecular changes in the periodontium. Indeed, LPS-induced cytokine responses were found to decline with age [41]. Fortin et al. showed that altered TREM-1 signaling in aged neutrophils was explained by defective recruitment of TREM-1 to lipid rafts [28], which may lead to altered shedding of sTREM-1 in the elderly. Therefore, the similar sTREM-1 levels seen among clinical groups in the present elderly population are likely to be related to these phenomena.

Less is known about the relationship of sTREM-1 and periodontal pathogens. In vitro studies have shown that *P. gingivalis* induces TREM-1 production in monocytes and secretion of TREM-1 in its soluble form [24, 26, 29]. In contrast, it was recently demonstrated in a mouse study that host immune responses to *P. gingivalis*, including TREM-1 expression, are reduced by age [42]. Another explanation could be that differences in dietary habits, increased flow of GCF by inflamed gingiva, and possible age-related changes in salivary gland secretions may similarly alter the conditions for growth and multiplication of the plaque-associated bacteria [43, 44]. Hence, the low differences in bacterial populations, with the exception of *T. forsythia*, at a given site may result in a similar inflammatory profile, including sTREM-1, between the healthy and diseased groups observed here.

The potential limitation of this study is that it is cross-sectional in nature, and thus does not allow for the continuous monitoring of the studied inflammatory mediator over time in this patient cohort. Future studies could address a similar question on sTREM-1 in a prospective manner, monitoring patients of different ages and over longer periods of time.

## Conclusions

The present study demonstrates that in the instance of periodontal disease, GCF sTREM levels are not elevated in the elderly population, and that only limited differences exist in their studied subgingival microbial profiles. Hence, these findings are in line with the notion that the severity of periodontitis in older age could result simply from the cumulative inflammatory effect of prolonged exposure to microbial challenge [45].

## Abbreviations

ANOVA: Analysis of variance
BOP: Bleeding on probing
CAL: Clinical attachment level
ELISA: Enzyme-linked immunosorbent assay
GCF: Gingival crevicular fluid, CP, chronic periodontitis
PI: Plaque index
PPD: Probing-pocket depth
qPCR: Quantitative real-time polymerase chain reaction
TREM-1: Triggering receptor expressed on myeloid cells 1
